# Disseminated Extrapulmonary Tuberculosis in an Immunocompetent Individual Without Pulmonary Involvement: A Case Report

**DOI:** 10.7759/cureus.64138

**Published:** 2024-07-09

**Authors:** Mohammed A AlHajji, Ahmed Auda, Abdulazez K Almukhizem, Ghadeer A Al ibraheem, Hawra A Alqassab, Ghadeer R Alkhouder

**Affiliations:** 1 General Surgery, Al Ahsa Health Cluster, Al Ahsa, SAU; 2 General and Laparoscopic Surgery, Al Ahsa Health Cluster, Al Ahsa, SAU; 3 Radiology, Al Ahsa Health Cluster, Al Ahsa, SAU; 4 Medicine, King Faisal University, Al Ahsa, SAU; 5 Wound Care, Al Ahsa Health Cluster, Al Ahsa, SAU

**Keywords:** from india, paraspinal tuberculosis, brain tuberculoma, disseminated tuberculosis, cold abscess, immunocompetent indivisuals, extrapulmonary tb, tubercolosis

## Abstract

Tuberculosis (TB) is a major global health burden, particularly in developing countries like India. While the most common presentation is pulmonary TB, extrapulmonary TB involving other body systems can also occur, posing diagnostic challenges. We present the case of a 24-year-old immunocompetent man from India who exhibited an uncommon and complex presentation of disseminated extrapulmonary TB. The patient had an asymptomatic brain cavitated lesion, likely tuberculoma, cervical lymphadenopathy, a small subcutaneous collection in the neck, a destructive lytic lesion in the sacrum, and a subcutaneous collection in the left gluteal/paraspinal region, all in the absence of pulmonary involvement. This combination of manifestations has not been previously reported. The presence of cervical lymphadenopathy and a slowly growing subcutaneous abscess were important clues that guided the diagnostic workup. Maintaining a high index of suspicion for TB, even in atypical presentations and immunocompetent individuals, is crucial, particularly in high-TB-burden regions. This case highlights the importance of considering disseminated extrapulmonary TB in the differential diagnosis, even in the absence of pulmonary involvement and typical risk factors. A high index of suspicion, a multidisciplinary approach, and a comprehensive diagnostic workup are essential for the timely recognition and management of these challenging conditions.

## Introduction

Tuberculosis (TB) is a serious infectious disease caused by the bacterium *Mycobacterium tuberculosis*. The global burden of TB is significant, with the World Health Organization (WHO) reporting 10 million new cases and 1.5 million deaths worldwide in 2019 [1].

Geographically, TB is more prevalent in developing countries, particularly in Asia and Africa. India accounts for a significant portion of the global TB burden, with an estimated 2.6 million new cases reported in 2019 [1].

The most common mode of TB presentation is pulmonary TB, which affects the lungs. However, TB can also manifest in less common extrapulmonary forms, involving other body parts such as the lymph nodes, bones, joints, and the central nervous system [2]. Extrapulmonary TB can be challenging to diagnose, as the symptoms may be non-specific.

Overall, the global burden of TB remains a significant public health concern, with India being one of the countries most affected. Understanding the epidemiology and modes of presentation, including the potential for extrapulmonary involvement, is crucial for effective prevention, diagnosis, and management strategies.

## Case presentation

The patient is a 24-year-old Indian male farmer who began working in Saudi Arabia four months ago. He has no known chronic medical conditions. He presented with a history of progressive left paraspinal lumbar swelling for two months, accompanied by dull, continuous pain that has worsened over time. The pain is not radiating and does not change with position. There was no fever, skin changes, weight loss, recent contact with ill patients, respiratory symptoms, mental status changes, abdominal pain, urinary symptoms, or changes in bowel habits. All four limbs had intact neuromotor function with no complaints of numbness, weakness, or pain.

**Figure 1:**
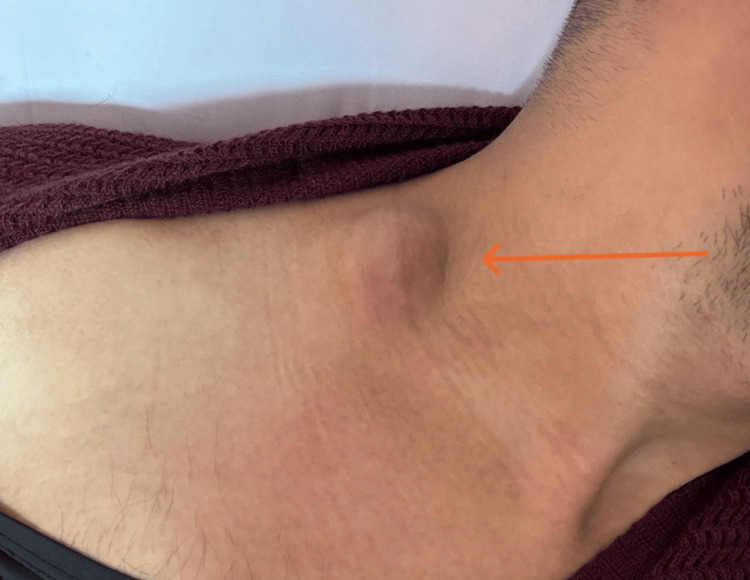
2.5 cm x 2.4 cm cystic lesion noted in the subcutaneous right lower neck.

**Figure 2:**
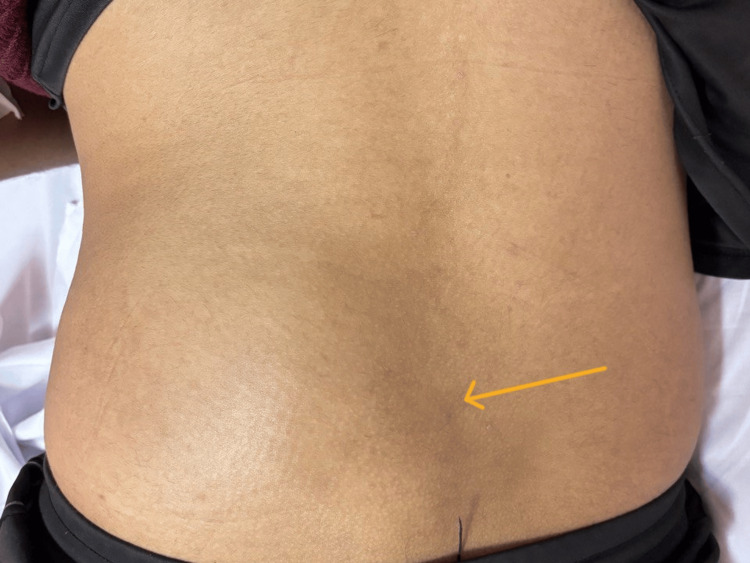
Left paraspinal and gluteal subcutaneous slowly progressive swelling from accumulated fluid (cold abscess).

The patient's past medical and surgical history is unremarkable, and he has no known history of allergies. On physical examination, his vital signs were within normal limits; he was conscious, alert, and oriented to time, place, and person. He was well-built and not cachectic. The head examination was unremarkable, but the neck examination revealed multiple small cervical lymph nodes and a small right cervical collection around 2 cm × 2 cm (Figure 1), with no surrounding elements of cellulitis. Back examination revealed a large, lower left paraspinal mass extending to the left gluteal region (Figure 2). The mass was fluctuant and mildly tender to palpation, but no specific spinal tenderness existed. The chest, abdomen, groin, and genitalia were clinically unremarkable.

Diagnostic testing included a complete blood count and biochemistry tests, which were unremarkable, as well as negative HIV and hepatitis profile testing. Ultrasound was done to assess the nature of the paraspinal fluid collection, followed by computed tomography (CT) of the cervical, chest, abdomen, and pelvis regions to further delineate the lesions and possible location of disseminated tuberculosis.

**Figure 3:**
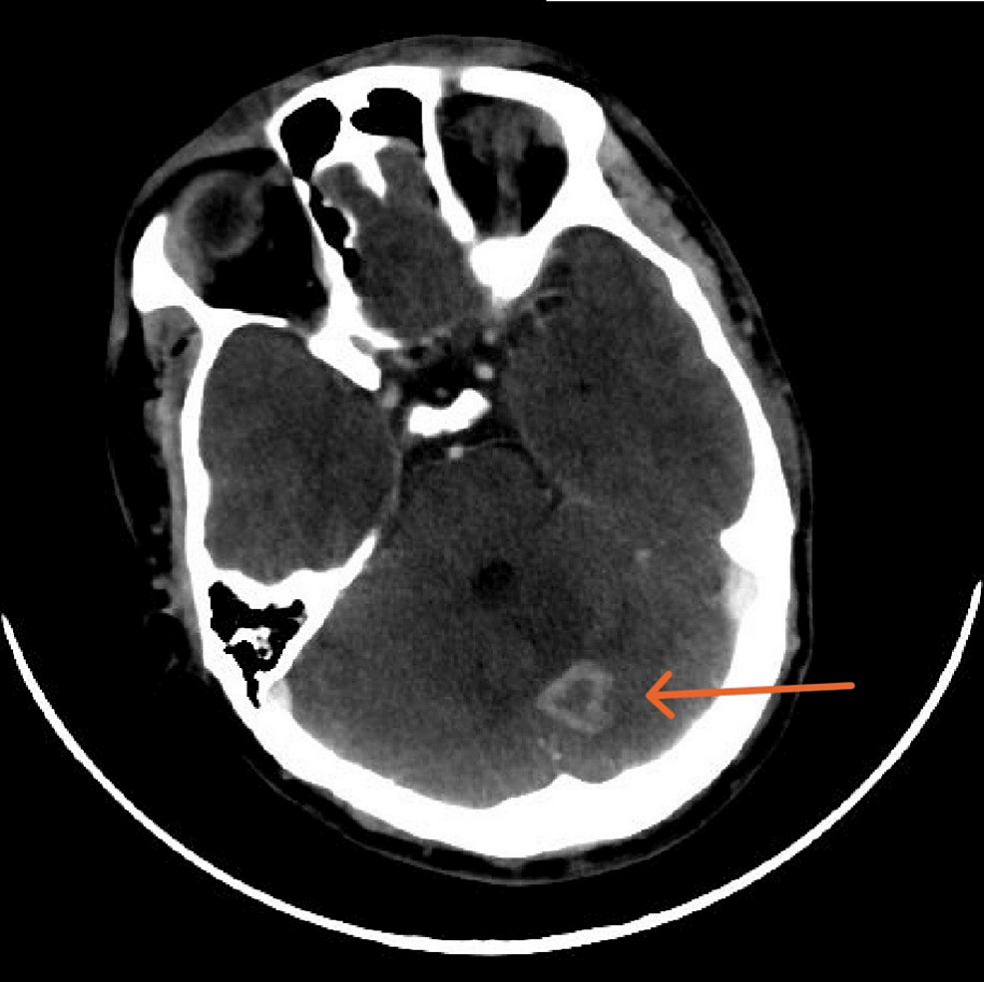
Axial cut CT of the brain demonstrating 2.2 cm × 2.3 cm ring enhancing left cerebellar lesion with surrounding edema represents tuberculoma.

**Figure 4:**
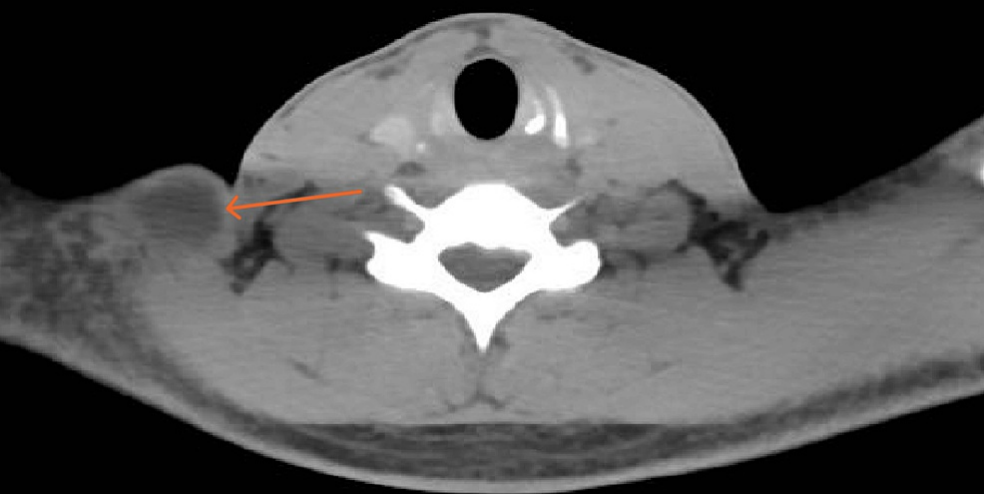
Axial cut CT of the neck demonstrates 2.5 cm × 2.4 cm cystic lesion noted in the subcutaneous right lower neck. No septation, No calcification.

**Figure 5:**
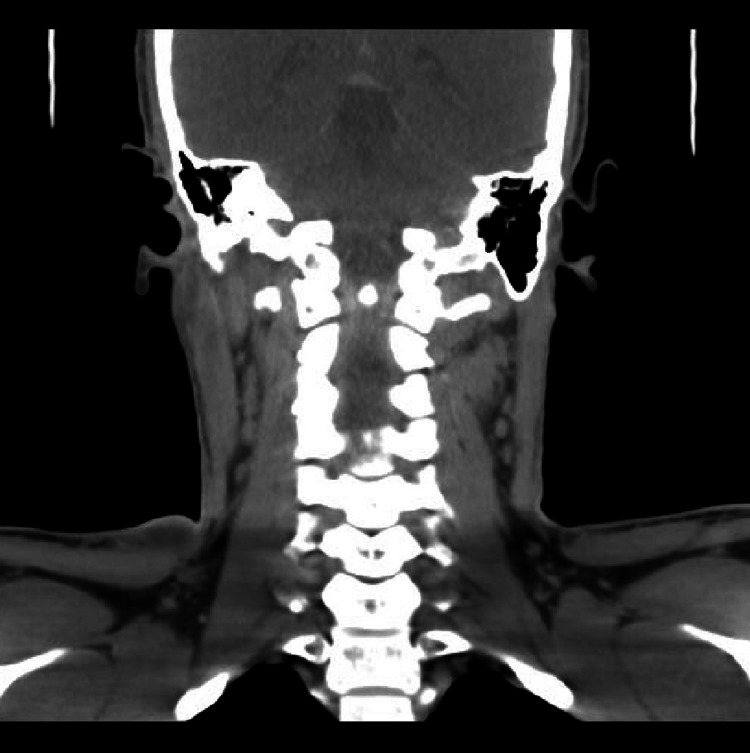
Coronal cut CT of the Neck showing multiple enlarged cervical lymph nodes.

**Figure 6:**
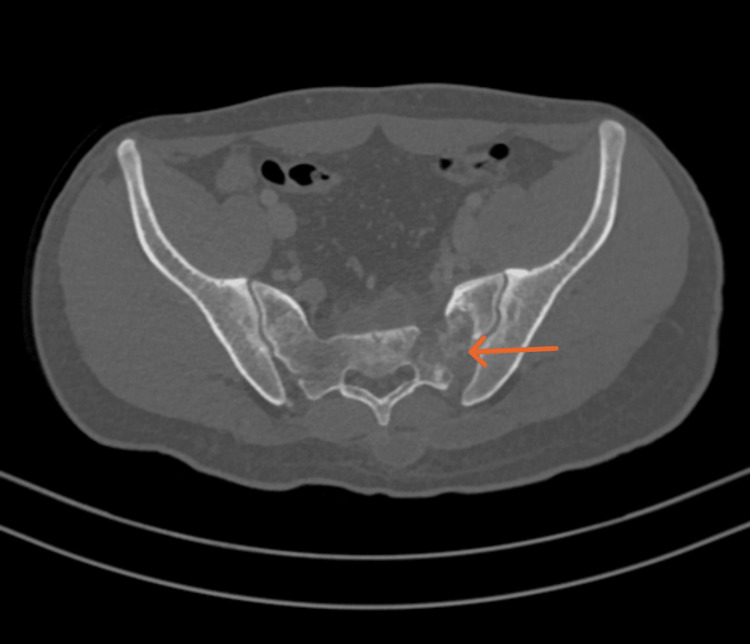
Axial cut CT of the pelvis demonstrating destructive lytic lesion in the left sacral bone.

**Figure 7:**
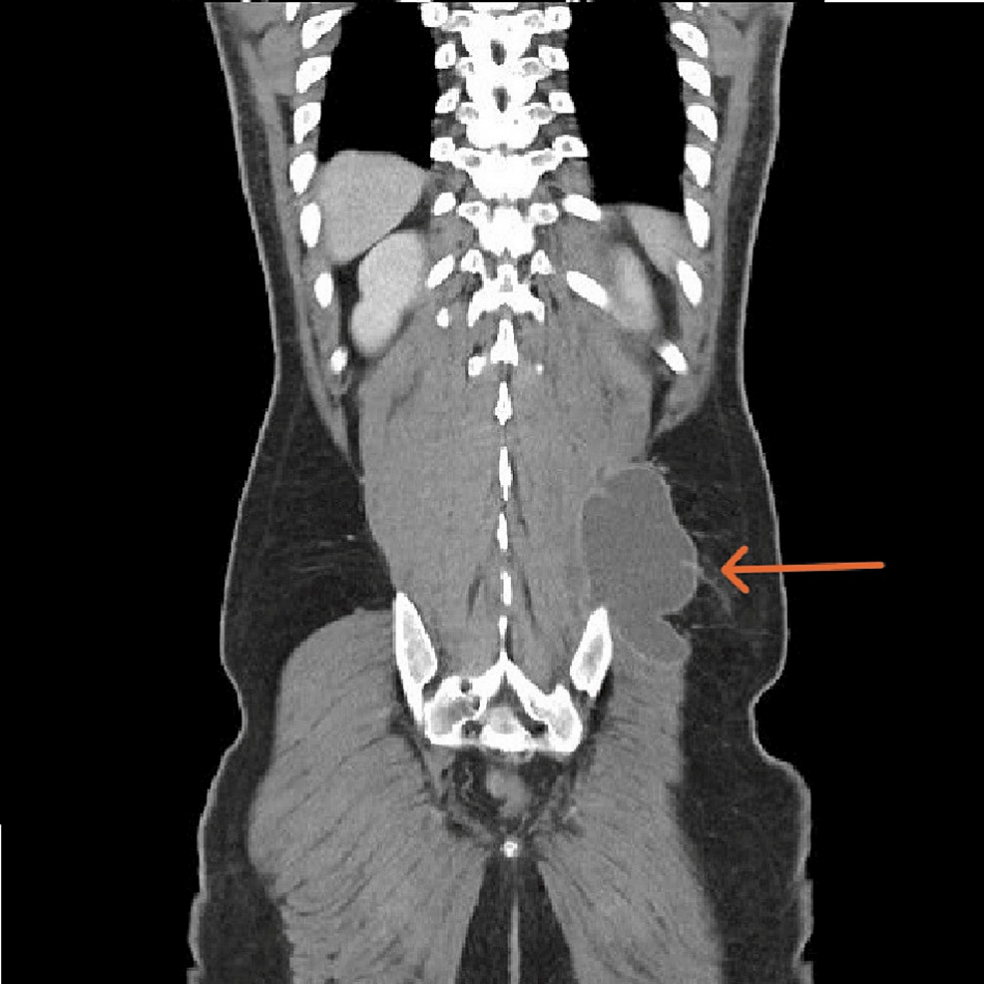
Coronal cut CT of the abdomen showing left gluteal/paraspinal collection measures approximately 12 cm × 6.5 cm, with anterior and posterior vertebral extension.

**Figure 8:**
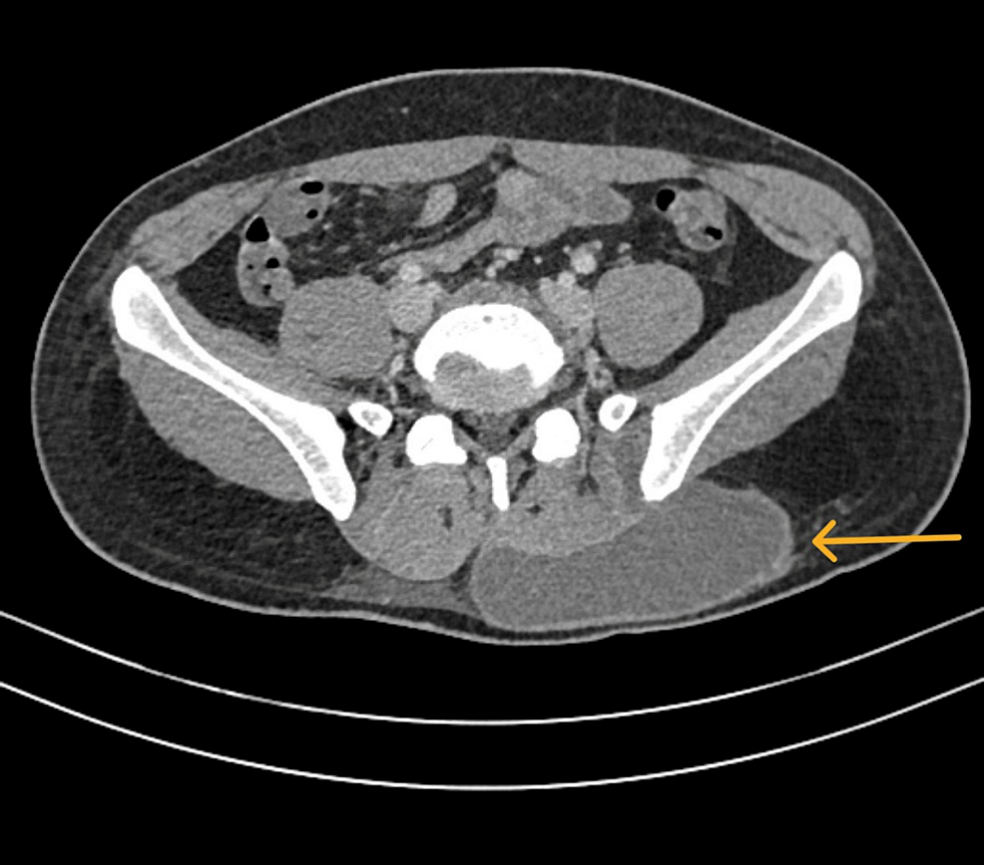
Axial cut CT of the abdomen showing left gluteal/paraspinal collection measures approximately 12 cm × 6.5 cm, with anterior and posterior vertebral extension.

The CT findings revealed a 2.2 cm × 2.3 cm ring-enhancing left cerebellar lesion with surrounding edema suspicious for tuberculoma (Figure 3), a 2.5 cm × 2.4 cm cystic lesion in the subcutaneous right lower neck with no septation or calcification, and multiple enlarged cervical lymph nodes (Figures 4-5). In the abdomen and pelvis, there was a destructive lytic lesion in the left sacral bone (Figure 6), associated with a left gluteal/paraspinal collection measuring approximately 12 cm × 6.5 cm, with anterior and posterior vertebral extension, but no septation or calcification (Figures 7-8). The chest CT was negative for any suspicious lesions.

An interventional radiologist was consulted, and a drainage catheter was placed in the left gluteal/paraspinal collection. Approximately 300 cc of purulent pus was evacuated, and the drain was kept in place for four days until the output was less than 20 cc per 24 hours for more than two consecutive days. The drained pus collection tested positive for Mycobacterium tuberculosis by the TB PCR test method and was negative for aerobic, anaerobic, and fungal infections.

After the drainage procedure, the patient was referred to the infectious disease service to plan and initiate anti-tuberculosis (anti-TB) therapy. The patient was instructed to continue follow-up care as recommended by the infectious disease specialists.

## Discussion

Disseminated extrapulmonary TB is an uncommon presentation, representing only 15-20% of global TB cases [1]. Even more rare in this disseminated extrapulmonary TB presentation are the specific findings of brain involvement and subcutaneous abscess. Brain tuberculosis, also called neurotuberculosis, is observed in less than 1% of all TB cases [3,4]. On the other hand, subcutaneous abscesses are an exceptionally uncommon extrapulmonary manifestation of tuberculosis, with only a small number of case reports documented in the literature [5,6].

In this case presentation, the patient exhibited an asymptomatic brain cavitated lesion, likely tuberculoma, along with cervical lymphadenopathy, a small subcutaneous collection in the neck, a destructive lytic lesion in the sacrum, and a subcutaneous collection in the left gluteal/paraspinal region - all in the absence of pulmonary involvement. A thorough review of the literature did not reveal any prior cases of extrapulmonary tuberculosis demonstrating this exact combination of manifestations, especially in immunocompetent individuals.

Cervical lymphadenopathy is a common presentation of extrapulmonary TB, seen in up to 35% of cases [7]. As observed in this patient, the involvement of cervical lymph nodes and a cold, slow-growing abscess for months is often the initial manifestation of disseminated disease and can be an important clue to guide the diagnostic workup.

In cases of disseminated extrapulmonary tuberculosis, the lymphatic system is commonly affected due to the bloodborne spread of *M. tuberculosis*. This can lead to the enlargement and caseation of lymph nodes, particularly in the cervical region. The involvement of the lymph nodes can result in the development of cold abscesses, sinus tracts, and fistulas. These atypical manifestations in the lymphatic system pose diagnostic challenges, as the clinical presentation may mimic other infectious or non-infectious conditions [8,9].

The presence of these atypical findings in this otherwise immunocompetent patient highlights the clinical challenge of diagnosing disseminated extrapulmonary TB, as the presentation can mimic a variety of other infectious and non-infectious conditions. Clinicians must maintain a high index of suspicion for TB, especially for a population considered a high endemic area for tuberculosis, even in the absence of typical pulmonary involvement and immunocompatibility status, to ensure timely diagnosis and management.

The diagnostic approach can vary depending on the clinical presentation. It may involve a combination of different imaging studies, such as radiography, computed tomography, or magnetic resonance imaging. Microbiological testing methods, including acid-fast bacilli (AFB) smears, mycobacterial culture, and molecular assays, may also be utilized. Additionally, histopathological examination of the affected tissues may be performed in cases of diagnosis uncertainty [2]. Careful examination and appropriate sampling of these lesions can provide valuable microbiological and histopathological information to support the diagnosis of disseminated TB.

In the presented case, we started with ultrasonography of the subcutaneous lesion as the initial step to give us a clue about the nature of this swelling. We then moved to computed tomography of the neck, chest, abdomen, and pelvis after developing a high clinical suspicion of tuberculosis based on the previously mentioned findings. We chose to sample and drain the subcutaneous collection, as it was the most symptomatically significant for the patient, and due to the technically easier access, we avoided a more invasive and morbid procedure. We finally confirmed our top differential diagnosis of tuberculosis through microbiology testing of the paraspinal subcutaneous abscess collection using the drainage catheter.

The main treatment for disseminated extrapulmonary tuberculosis is medical, following the same principles as the treatment for pulmonary tuberculosis. The standard regimen is a six-month course of four first-line anti-tuberculosis drugs: isoniazid, rifampicin, pyrazinamide, and ethambutol. However, the duration of treatment may need to be extended in cases of extensive disease or a slow clinical response [2].

In cases with specific complications, such as symptomatic brain tuberculomas or subcutaneous abscesses, adjunctive surgical intervention may be necessary, depending on the individual assessment of each case. This may include drainage of abscesses, debridement of necrotic tissue, or excision of tuberculomas if there are space-occupying consequences. The need for surgical management should be determined on a case-by-case basis as part of the overall multidisciplinary approach to managing these complex presentations of disseminated extrapulmonary tuberculosis [3-6].

Close monitoring and a multidisciplinary approach involving specialists like infectious disease specialists, radiologists, surgeons, and other areas of interest depending on the clinical presentation of each case are crucial for the successful management of these complex cases of disseminated extrapulmonary tuberculosis.

## Conclusions

This case illustrates the rare presentation of disseminated extrapulmonary tuberculosis, with unusual findings of an asymptomatic brain tuberculoma and a subcutaneous abscess in an otherwise immunocompetent patient. The presence of a slowly growing abscess (cold abscess) and cervical lymphadenopathy were important clues that guided the diagnostic workup. Maintaining a high index of suspicion for tuberculosis, even in atypical presentations or immunocompatibility status, and employing a comprehensive diagnostic approach are essential for the timely recognition and management of this challenging condition, especially in high tuberculosis burden regions.
